# *N*-Cyclo­hexyl-2-(2,3-dichloro­phenyl­sulfan­yl)acetamide

**DOI:** 10.1107/S1600536808040464

**Published:** 2008-12-06

**Authors:** Zhu-Bo Li, Jing Li, Wen-Liang Dong, Hua Zuo, Xiao-Yan He

**Affiliations:** a College of Pharmaceutical Sciences, Southwest University, Chongqing 400716, People’s Republic of China; b Shandong University of Traditional Chinese Medicine, Jinan 250355, People’s Republic of China

## Abstract

In the crystal structure of title compound, C_14_H_17_Cl_2_NOS, the cyclo­hexyl ring has a chair conformation and connects with an equatorial N atom. Mol­ecules are connected *via* N—H⋯O hydrogen bonds into chains.

## Related literature

For related literature, see: Li *et al.* (2008*a* ▶,*b* ▶).
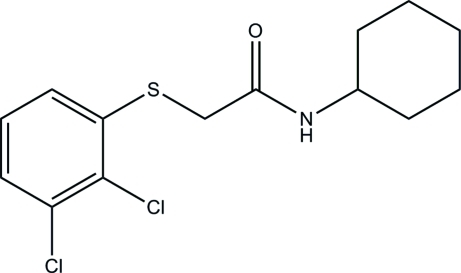


## Experimental

### 

#### Crystal data

C_14_H_17_Cl_2_NOS
*M* *_r_* = 318.25
Monoclinic, 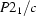

*a* = 13.427 (2) Å
*b* = 12.877 (2) Å
*c* = 9.1807 (16) Å
β = 104.849 (3)°
*V* = 1534.3 (5) Å^3^
*Z* = 4
Mo *K*α radiation
μ = 0.55 mm^−1^
*T* = 293 (2) K
0.10 × 0.06 × 0.02 mm

#### Data collection

Bruker SMART CCD area-detector diffractometer
Absorption correction: multi-scan (*SADABS*; Bruker, 2005 ▶) *T*_min_ = 0.947, *T*_max_ = 0.989
7968 measured reflections
2712 independent reflections
1972 reflections with *I* > 2σ(*I*)
*R*_int_ = 0.033

#### Refinement

*R*[*F* ^2^ > 2σ(*F* ^2^)] = 0.038
*wR*(*F* ^2^) = 0.095
*S* = 1.05
2712 reflections
173 parameters
H-atom parameters constrained
Δρ_max_ = 0.26 e Å^−3^
Δρ_min_ = −0.27 e Å^−3^

### 

Data collection: *SMART* (Bruker, 2005 ▶); cell refinement: *SAINT* (Bruker, 2005 ▶); data reduction: *SAINT* (Bruker, 2005 ▶); program(s) used to solve structure: *SHELXS97* (Sheldrick, 2008 ▶); program(s) used to refine structure: *SHELXL97* (Sheldrick, 2008 ▶); molecular graphics: *SHELXTL* (Sheldrick, 2008 ▶); software used to prepare material for publication: *SHELXTL*.

## Supplementary Material

Crystal structure: contains datablocks I, global. DOI: 10.1107/S1600536808040464/cs2098sup1.cif

Structure factors: contains datablocks I. DOI: 10.1107/S1600536808040464/cs2098Isup2.hkl

Additional supplementary materials:  crystallographic information; 3D view; checkCIF report

## Figures and Tables

**Table 1:** Hydrogen-bond geometry (Å, °)

*D*—H⋯*A*	*D*—H	H⋯*A*	*D*⋯*A*	*D*—H⋯*A*
N—H0*A*⋯O2^i^	0.86	2.01	2.867 (2)	177

Symmetry code: (i) 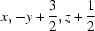.

**Fig. 1.**
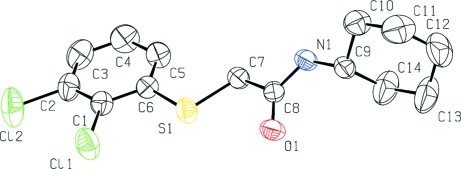
The molecular structure of the title compound showing displacement ellipsoids drawn at 50% probability level. H atoms are omitted for clarity.

## supplementary crystallographic information

### Comment

The structure determination was performed as a part of a project on the interactions of small molecules with proteins. The structures of the similar compounds *N*-benzyl-2-(2-chloro-4-methylphenoxy)acetamide (Li *et al.*, 2008*a*) and *N*-benzyl-2-(2,6-dichlorophenoxy)acetamide (Li *et al.*, 2008*b*) were reported previously.

In the crystal structure the cyclohexyl ring is in a chair conformation. The molecules are connected *via* N—H···O hydrogen bonding between the N—H H atom and the carbonyl O atom into chains, that extend in the direction of the *c* axis.

### Experimental

The solution of 2,3-dichlorobenzenethiol (1.0 mmol), *N*-cyclohexyl-2-chloroacetamide (1.1 mmol), K_2_CO_3_ (1.1 mmol) and CH_3_CN (20 ml) was refluxed for 4 h. After completion of the reaction (by TLC monitoring), the solution was cooled and solvent was evaporated under reduced pressure. The residue was poured into water and adjusted the pH 6–7 with dilute hydrochloric acid (10%) and extracted with ethyl acetate, washed with brine and dried over anhydrous MgSO_4_ to obtain the corresponding crude product. The product was purified by column chromatography on silica gel using ethyl acetate as eluent (yield 80%). Crystals suitable for X-ray diffraction were obtained by slow evaporation of a solution of the solid dissolved in ethyl acetate/hexane at room temperatures for 6 d.

### Refinement

All H atoms were placed in geometrically calculated positions and refined using a riding model with C—H = 0.97 Å (for CH_2_ groups).

### Crystal data

**Table:**

C_14_H_17_Cl_2_NOS	*F*(000) = 664
*M**_r_* = 318.25	*D*_x_ = 1.378 Mg m^−^^3^
Monoclinic, *P*2_1_/*c*	Mo *K*α radiation, λ = 0.71073 Å
Hall symbol: -P 2ybc	Cell parameters from 1963 reflections
*a* = 13.427 (2) Å	θ = 2.8–23.3°
*b* = 12.877 (2) Å	µ = 0.55 mm^−^^1^
*c* = 9.1807 (16) Å	*T* = 293 K
β = 104.849 (3)°	Needle, colourless
*V* = 1534.3 (5) Å^3^	0.10 × 0.06 × 0.02 mm
*Z* = 4	

### Data collection

**Table:**

Bruker SMART CCD area-detector diffractometer	2712 independent reflections
Radiation source: fine-focus sealed tube	1972 reflections with *I* > 2σ(*I*)
graphite	*R*_int_ = 0.033
φ and ω scans	θ_max_ = 25.1°, θ_min_ = 1.6°
Absorption correction: multi-scan (*SADABS*; Bruker, 2005)	*h* = −15→15
*T*_min_ = 0.947, *T*_max_ = 0.989	*k* = −15→15
7968 measured reflections	*l* = −10→10

### Refinement

**Table:**

Refinement on *F*^2^	Secondary atom site location: difference Fourier map
Least-squares matrix: full	Hydrogen site location: inferred from neighbouring sites
*R*[*F*^2^ > 2σ(*F*^2^)] = 0.038	H-atom parameters constrained
*wR*(*F*^2^) = 0.095	*w* = 1/[σ^2^(*F*_o_^2^) + (0.0364*P*)^2^ + 0.4415*P*] where *P* = (*F*_o_^2^ + 2*F*_c_^2^)/3
*S* = 1.05	(Δ/σ)_max_ = 0.001
2712 reflections	Δρ_max_ = 0.26 e Å^−^^3^
173 parameters	Δρ_min_ = −0.27 e Å^−^^3^
0 restraints	Extinction correction: *SHELXL97* (Sheldrick, 2008), Fc^*^=kFc[1+0.001xFc^2^λ^3^/sin(2θ)]^-1/4^
Primary atom site location: structure-invariant direct methods	Extinction coefficient: 0.0083 (12)

### Fractional atomic coordinates and isotropic or equivalent isotropic displacement parameters (Å^2^)

**Table:**

	*x*	*y*	*z*	*U*_iso_*/*U*_eq_	
S1	0.85908 (5)	0.72227 (5)	0.05020 (7)	0.0493 (2)	
Cl1	1.06216 (5)	0.73736 (6)	−0.03167 (9)	0.0719 (3)	
Cl2	1.24245 (6)	0.58238 (7)	0.09765 (11)	0.0941 (3)	
O1	0.66239 (12)	0.81810 (13)	−0.02975 (18)	0.0506 (4)	
C1	1.04846 (17)	0.64033 (18)	0.0911 (3)	0.0455 (6)	
C2	1.12759 (19)	0.5724 (2)	0.1488 (3)	0.0549 (7)	
C3	1.1172 (2)	0.4955 (2)	0.2479 (3)	0.0647 (8)	
H3	1.1712	0.4502	0.2875	0.078*	
C4	1.0261 (2)	0.4870 (2)	0.2872 (3)	0.0639 (7)	
H4	1.0184	0.4352	0.3541	0.077*	
C5	0.94551 (19)	0.55371 (19)	0.2295 (3)	0.0528 (6)	
H5	0.8838	0.5460	0.2567	0.063*	
C6	0.95562 (17)	0.63200 (17)	0.1317 (3)	0.0409 (5)	
C7	0.75922 (16)	0.69227 (18)	0.1412 (3)	0.0436 (6)	
H7A	0.7322	0.6232	0.1129	0.052*	
H7B	0.7862	0.6945	0.2498	0.052*	
C8	0.67521 (16)	0.77252 (17)	0.0911 (3)	0.0395 (5)	
N1	0.61760 (14)	0.78834 (15)	0.1866 (2)	0.0483 (5)	
H1	0.6331	0.7557	0.2711	0.058*	
C9	0.52927 (17)	0.85822 (19)	0.1558 (3)	0.0461 (6)	
H9	0.5418	0.9135	0.0893	0.055*	
C10	0.5189 (2)	0.9077 (2)	0.2997 (3)	0.0596 (7)	
H10A	0.5807	0.9472	0.3443	0.072*	
H10B	0.5121	0.8539	0.3704	0.072*	
C11	0.4258 (3)	0.9791 (3)	0.2719 (4)	0.0849 (10)	
H11A	0.4185	1.0055	0.3676	0.102*	
H11B	0.4368	1.0379	0.2118	0.102*	
C12	0.3288 (3)	0.9245 (3)	0.1922 (4)	0.0917 (11)	
H12A	0.2721	0.9736	0.1706	0.110*	
H12B	0.3133	0.8707	0.2571	0.110*	
C13	0.3393 (2)	0.8767 (3)	0.0481 (4)	0.0904 (11)	
H13A	0.3477	0.9311	−0.0210	0.108*	
H13B	0.2772	0.8383	0.0015	0.108*	
C14	0.4319 (2)	0.8039 (3)	0.0773 (4)	0.0812 (10)	
H14A	0.4203	0.7460	0.1386	0.097*	
H14B	0.4389	0.7763	−0.0178	0.097*	

### Atomic displacement parameters (Å^2^)

**Table:**

	*U*^11^	*U*^22^	*U*^33^	*U*^12^	*U*^13^	*U*^23^
S1	0.0419 (3)	0.0507 (4)	0.0586 (4)	0.0076 (3)	0.0191 (3)	0.0136 (3)
Cl1	0.0579 (4)	0.0733 (5)	0.0933 (6)	0.0049 (3)	0.0355 (4)	0.0264 (4)
Cl2	0.0492 (5)	0.1011 (7)	0.1388 (8)	0.0170 (4)	0.0364 (5)	0.0111 (6)
O1	0.0577 (10)	0.0588 (10)	0.0387 (9)	0.0133 (8)	0.0183 (8)	0.0075 (8)
C1	0.0441 (13)	0.0426 (14)	0.0500 (15)	0.0009 (11)	0.0124 (11)	−0.0024 (12)
C2	0.0416 (14)	0.0547 (16)	0.0675 (18)	0.0065 (12)	0.0124 (12)	−0.0051 (14)
C3	0.0586 (17)	0.0533 (17)	0.076 (2)	0.0156 (14)	0.0058 (15)	0.0047 (15)
C4	0.0727 (19)	0.0484 (16)	0.0700 (19)	0.0114 (14)	0.0175 (15)	0.0152 (14)
C5	0.0537 (15)	0.0471 (14)	0.0599 (16)	0.0036 (12)	0.0189 (13)	0.0064 (13)
C6	0.0425 (13)	0.0366 (12)	0.0431 (13)	0.0022 (10)	0.0100 (10)	−0.0015 (11)
C7	0.0432 (13)	0.0476 (14)	0.0417 (13)	0.0029 (11)	0.0142 (10)	0.0024 (11)
C8	0.0374 (12)	0.0429 (13)	0.0383 (13)	−0.0024 (10)	0.0101 (10)	−0.0063 (11)
N1	0.0471 (11)	0.0620 (13)	0.0394 (11)	0.0143 (10)	0.0174 (9)	0.0104 (10)
C9	0.0431 (13)	0.0552 (15)	0.0429 (14)	0.0087 (11)	0.0165 (11)	0.0060 (12)
C10	0.0648 (17)	0.0590 (17)	0.0545 (16)	0.0130 (14)	0.0140 (14)	−0.0059 (14)
C11	0.100 (3)	0.084 (2)	0.070 (2)	0.044 (2)	0.0194 (19)	−0.0107 (18)
C12	0.068 (2)	0.113 (3)	0.105 (3)	0.034 (2)	0.042 (2)	0.009 (2)
C13	0.0447 (17)	0.106 (3)	0.111 (3)	0.0128 (17)	0.0022 (17)	−0.025 (2)
C14	0.0518 (17)	0.088 (2)	0.095 (2)	0.0106 (16)	0.0030 (16)	−0.0380 (19)

### Geometric parameters (Å, °)

**Table:**

S1—C6	1.759 (2)	N1—H1	0.8600
S1—C7	1.794 (2)	C9—C14	1.495 (4)
Cl1—C1	1.724 (2)	C9—C10	1.505 (3)
Cl2—C2	1.728 (3)	C9—H9	0.9800
O1—C8	1.228 (2)	C10—C11	1.520 (4)
C1—C2	1.373 (3)	C10—H10A	0.9700
C1—C6	1.394 (3)	C10—H10B	0.9700
C2—C3	1.376 (4)	C11—C12	1.496 (4)
C3—C4	1.365 (4)	C11—H11A	0.9700
C3—H3	0.9300	C11—H11B	0.9700
C4—C5	1.377 (3)	C12—C13	1.499 (4)
C4—H4	0.9300	C12—H12A	0.9700
C5—C6	1.380 (3)	C12—H12B	0.9700
C5—H5	0.9300	C13—C14	1.524 (4)
C7—C8	1.512 (3)	C13—H13A	0.9700
C7—H7A	0.9700	C13—H13B	0.9700
C7—H7B	0.9700	C14—H14A	0.9700
C8—N1	1.325 (3)	C14—H14B	0.9700
N1—C9	1.457 (3)		
			
C6—S1—C7	102.52 (11)	N1—C9—H9	108.0
C2—C1—C6	120.3 (2)	C14—C9—H9	108.0
C2—C1—Cl1	120.80 (19)	C10—C9—H9	108.0
C6—C1—Cl1	118.90 (18)	C9—C10—C11	111.5 (2)
C1—C2—C3	120.9 (2)	C9—C10—H10A	109.3
C1—C2—Cl2	120.3 (2)	C11—C10—H10A	109.3
C3—C2—Cl2	118.8 (2)	C9—C10—H10B	109.3
C4—C3—C2	118.9 (2)	C11—C10—H10B	109.3
C4—C3—H3	120.6	H10A—C10—H10B	108.0
C2—C3—H3	120.6	C12—C11—C10	111.9 (3)
C3—C4—C5	121.1 (3)	C12—C11—H11A	109.2
C3—C4—H4	119.4	C10—C11—H11A	109.2
C5—C4—H4	119.4	C12—C11—H11B	109.2
C4—C5—C6	120.5 (2)	C10—C11—H11B	109.2
C4—C5—H5	119.7	H11A—C11—H11B	107.9
C6—C5—H5	119.7	C11—C12—C13	110.9 (3)
C5—C6—C1	118.3 (2)	C11—C12—H12A	109.4
C5—C6—S1	125.10 (18)	C13—C12—H12A	109.4
C1—C6—S1	116.58 (17)	C11—C12—H12B	109.4
C8—C7—S1	107.41 (15)	C13—C12—H12B	109.4
C8—C7—H7A	110.2	H12A—C12—H12B	108.0
S1—C7—H7A	110.2	C12—C13—C14	110.7 (3)
C8—C7—H7B	110.2	C12—C13—H13A	109.5
S1—C7—H7B	110.2	C14—C13—H13A	109.5
H7A—C7—H7B	108.5	C12—C13—H13B	109.5
O1—C8—N1	123.6 (2)	C14—C13—H13B	109.5
O1—C8—C7	121.53 (19)	H13A—C13—H13B	108.1
N1—C8—C7	114.8 (2)	C9—C14—C13	111.7 (2)
C8—N1—C9	123.41 (19)	C9—C14—H14A	109.3
C8—N1—H1	118.3	C13—C14—H14A	109.3
C9—N1—H1	118.3	C9—C14—H14B	109.3
N1—C9—C14	111.9 (2)	C13—C14—H14B	109.3
N1—C9—C10	110.20 (19)	H14A—C14—H14B	107.9
C14—C9—C10	110.7 (2)		
			
S1—C7—C8—O1	25.9 (3)	C14—C9—C10—C11	−54.3 (3)
S1—C7—C8—N1	−154.55 (17)	C9—C10—C11—C12	54.7 (4)
O1—C8—N1—C9	3.0 (4)	C10—C11—C12—C13	−55.3 (4)
C7—C8—N1—C9	−176.6 (2)	C11—C12—C13—C14	55.8 (4)
C8—N1—C9—C14	89.3 (3)	N1—C9—C14—C13	178.9 (3)
C8—N1—C9—C10	−147.0 (2)	C10—C9—C14—C13	55.5 (3)
N1—C9—C10—C11	−178.6 (2)	C12—C13—C14—C9	−56.5 (4)

### Hydrogen-bond geometry (Å, °)

**Table:**

*D*—H···*A*	*D*—H	H···*A*	*D*···*A*	*D*—H···*A*
N—H0A···O2^i^	0.86	2.01	2.867 (2)	177

Symmetry codes: (i) *x*, −*y*+3/2, *z*+1/2.
